# Diagnosis and treatment of community-associated methicillin-resistant *Staphylococcus aureus* prostatic abscess involving the seminal vesicle: A case report

**DOI:** 10.3892/etm.2014.2147

**Published:** 2014-12-18

**Authors:** XIANGYI ZHENG, XIAO WANG, JIN ZHOU, JIANJIAN XIANG, LIPING XIE

**Affiliations:** 1 Department of Urology, The First Affiliated Hospital, Zhejiang University School of Medicine, Hangzhou, Zhejiang 310003, P.R. China; 2 Department of Ultrasound, The First Affiliated Hospital, Zhejiang University School of Medicine, Hangzhou, Zhejiang 310003, P.R. China

**Keywords:** ultrasound perfusion imaging, prostate needle aspiration, community-associated methicillin-resistant *Staphylococcus aureus*, prostatic abscess

## Abstract

Prostatic abscess involving the seminal vesicle is a rare disease due to the widespread use of broad-spectrum antibiotics in patients with lower urinary tract symptoms. With the elevated risk of community-associated methicillin-resistant *Staphylococcus aureus* (CA-MRSA), a great emphasis on its pathogenicity in prostatic abscesses is required. To the best of our knowledge, this is the first study to describe the use of ultrasound perfusion imaging and traditional computed tomography to diagnose a patient with CA-MRSA prostate abscess involving the seminal vesicle. In the present case, the patient was successfully treated via adjuvant antibiotics and transrectal ultrasonography (TRUS)-guided prostate needle aspiration. Prostatic abscess involving the seminal vesicle is an uncommon disease with a potential risk of mortality if the appropriate treatment is not provided, and thus deserves particular attention. Ultrasound perfusion imaging plays an important role in the diagnosis of prostatic abscess involving the seminal vesicle. In the present case, early treatment with adjuvant antibiotics and TRUS-guided needle aspiration of the prostatic abscess resulted in a shorter hospital stay, and lower risk of local recurrence and mortality.

## Introduction

Methicillin-resistant *Staphylococcus aureus* (MRSA)-induced infections, accounting for >50% of staphylococcal diseases, have increased rapidly in recent years (1). MRSA is considered to be one of the most common antibiotic-resistant pathogens causing invasive infections (2). In the last decade, epidemiologic studies have illustrated that MRSA-induced infections have changed from being primarily acquired in hospitals to also being acquired in the community (3). Between 1999 and 2005, the estimated number of MRSA infections more than doubled in the United States (4). This increase was largely attributed to skin and soft-tissue infections, which are commonly caused by community-associated MRSA (CA-MRSA) (4).

Prostatic abscess is a rare disease due to the widespread use of broad-spectrum antibiotics in patients with lower urinary tract symptoms (LUTS) (5). It is mainly identified in patients with predisposing factors, including chronic indwelling catheters, invasion of the lower urinary tract, diabetes mellitus, human immunodeficiency virus infection and other causes of compromised immunity (6). In the past, prostatic abscesses were primarily caused by *Neisseria gonorrhoeae* and *Staphylococcus aureus*. At present, gram-negative bacteria, particularly *Escherichia coli*, are the dominant pathogens in the development of prostatic abscess (5). With the elevated risk of CA-MRSA, a great emphasis is put on its pathogenicity in prostatic abscess (7). Seminal vesicle abscess mostly results from concurrent prostatic abscess. Few cases have been reported since the first case in 1978 when Machida *et al* reported the first case of spermatic cord abscess with concurrent prostatic abscess involving the seminal vesicle (8).

To the best of our knowledge, the present study is the first to describe the use of ultrasound perfusion imaging and traditional computed tomography (CT) to diagnose a patient with CA-MRSA prostate abscess involving the seminal vesicle, which was subsequently treated with adjuvant antibiotics and transrectal ultrasonography (TRUS)-guided prostate needle aspiration. Written informed consent was obtained from the patient for publication of this case report and the accompanying images.

## Case report

**Figure 1:**
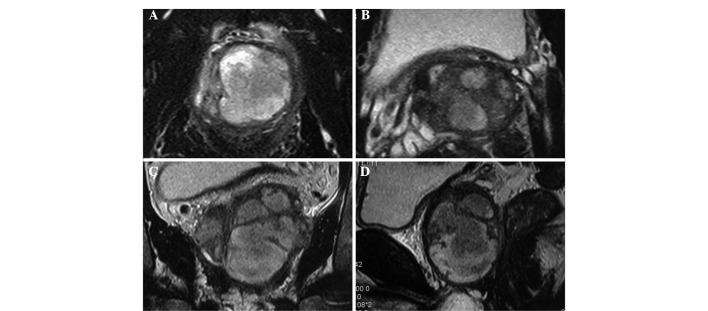
Magnetic resonance imaging exhibited an abnormal signal in the prostate and left seminal vesicle, indicating prostate abscesses involving the seminal vesicle. (A and B) Axial fat saturation images revealed multiple prostate abscesses involving the seminal vesicle; (C) coronal T2-weighted image; (D) sagittal T2-weighted image.

A 42-year-old male presented to the outpatient clinic of the First Affiliated Hospital of Zhejiang University School of Medicine (Hangzhou, China) with 2 weeks of severe dysuria and pain in the abdomen. The patient had been complaining of urination difficulty and dribbling for 4 years, but reported no nocturia, hematuria, fever or chills. The patient then went to the local hospital. Ultrasonography revealed a 5.6×3.9-cm abnormal echogenic mass in the prostate, which was considered to be an inflammatory mass. Magnetic resonance imaging (MRI) exhibited abnormal signals in the prostate and left seminal vesicle, indicating the presence of prostate abscesses involving the seminal vesicle (Fig. 1). Blood examinations revealed an elevated blood glucose level (18.62 mmol/l), and urine analysis demonstrated an elevated microscopic white cell count and the presence of glucose. Therefore, the patient was diagnosed with prostate abscesses involving the seminal vesicle, urine retention and diabetes mellitus (type 2). An indwelling catheter and anti-inflammatory and hypoglycemic therapy were administered in the local hospital. Eight days later, the catheter was removed. However, the patient continued to present with dysuria and odynuria. The patient also complained of constipation and persistent pain around the anus at this time. No significant past medical history was identified. Physical examination was remarkable due to the observation of an enlarged prostate, with tenderness.

**Figure 2:**
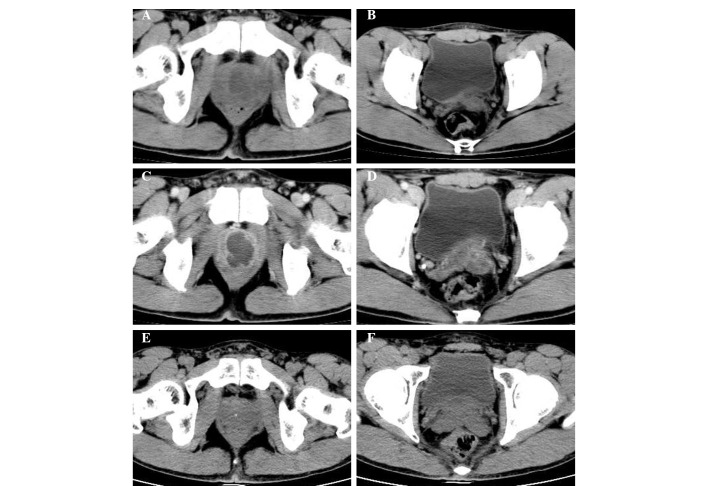
Pelvic computed tomography (CT) prior to and following prostate needle aspiration. (A and B) Noncontrast CT revealed multiple prostate abscesses, invading left seminal vesicle; (C and D) parenchymal phase CT images; (E and F) follow-up CT scans revealed a marked improvement in the prostate 1 month after the initial prostate needle aspiration.

**Figure 3:**
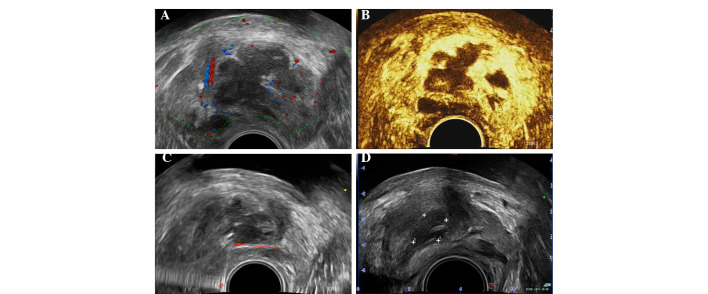
Ultrasound of the prostate prior to and following prostate needle aspiration. (A) Transrectal ultrasonography revealed multi-hypoechogenic masses in the prostate, which were considered to be abscesses; (B) transrectal ultrasound perfusion imaging illustrated no enhancement in the hypoechogenic area and irregular fluid dark space of the prostate; (C) puncture point of the prostate abscess; (D) transrectal ultrasonography revealed a great improvement in the prostate 4 days after prostate needle aspiration, with a 1.6×1.0-cm hypoechogenic area.

Subsequently, the patient was admitted to the Department of Urology, First Affiliated Hospital of Zhejiang University School of Medicine, where a series of examinations were conducted. Urine culture grew *Candida albicans* that was sensitive to fluconazole. Glycosylated hemoglobin analysis showed an elevation in glycosylated hemoglobin A1 (12.0%) and glycosylated hemoglobinA1c (10.5%) levels. The examination of a urine smear for *Mycobacterium tuberculosis* exhibited a negative result. CT of the pelvis demonstrated that there were multiple prostate abscesses, involving the left seminal vesicle (Fig. 2A–D). Transabdominal ultrasonography revealed multi-hypoechogenic masses in the prostate, which were considered to be abscesses. Transrectal ultrasonography was also conducted, which revealed similar results (Fig. 3A). Transrectal ultrasound perfusion imaging illustrated no enhancement in the hypoechogenic area and irregular fluid dark space of the prostate (Fig. 3B).

Therefore, the patient received levofloxacin, metronidazole and fluconazole for a total of 3 days before transrectal prostate puncture was performed (Fig. 3C). A total of 17 ml purulent fluid was extracted. Purulent fluid culture grew MRSA (sensitive to all antibiotics with the exception of penicillin, oxacillin and erythromycin), and levofloxacin and metronidazole were given for another 2 days prior to withdrawal. The patient was reexamined by transrectal ultrasonography, which revealed a great improvement in the prostate, with a 1.6×1.0-cm hypoechogenic area (Fig. 3D). The result of a C-reactive protein test was normal at 3.10 mg/l. The patient was discharged the next day.

A follow-up CT scan and ultrasonography 1 month after the initial presentation revealed a marked improvement in the prostate and the symptoms were greatly improved (Fig. 2E and F).

## Discussion

Prostatic abscess is considered to be an uncommon disease due to the widespread use of broad-spectrum antibiotics in patients with LUTS (5). Nevertheless, accurate diagnosis and treatment remain of great importance due to the possibility of progression to sepsis and mortality (9). Prostatic abscess is challenging to diagnose due to its non-specific signs, symptoms and physical examination findings, including fever, chills, urinary frequency, dysuria, acute urinary retention (AUR) or lower back pain, which is easily misdiagnosed as acute bacterial prostatitis (10). Imaging examination such as transrectal ultrasonography (TRUS), CT and MRI is critical for accurate diagnosis. TRUS can be applied as an initial examination and easily make a diagnosis of prostatic abscess. CT of the pelvis and abdomen area brings no extra pain to patients, and can help to confirm the scope of infection in the adjacent organs (11).

Seminal vesicle abscess usually results from concurrent prostatic abscess. The diagnosis is commonly made by CT or TRUS. The CT characteristics of a seminal vesicle abscess include: i) enlargement of the seminal vesicle unilaterally or bilaterally; ii) an area of low attenuation in the seminal vesicle; iii) inflammatory alterations in surrounding fat; and iv) thickening of the bladder wall focally or diffusely (12).

Ultrasound contrast can enhance the visualization of perfusion changes associated with prostate cancer, and is regarded as a promising tool in distinguishing between benign prostatic hyperplasia and prostate cancer (13,14). To the best of our knowledge, the present study is the first to report the use of ultrasound perfusion imaging and traditional CT to diagnose a patient with CA-MRSA prostate abscess involving the seminal vesicle. In this case, transrectal ultrasound perfusion imaging exhibited no enhancement in the hypoechogenic area and irregular fluid dark space of the prostate.

Treatments of prostatic abscess have changed greatly in recent years. Surgeries such as perineal incision or transurethral resection were traditionally recommended as the first-line therapy (15). By contrast, minimally invasive treatments such as TRUS-guided needle aspiration transrectally or transperineally under local anesthesia are more popular at present. Becker *et al* first reported that needle aspiration and adjuvant antibiotic therapy could achieve a superior therapeutic efficacy (16). Thereafter, many studies were conducted to evaluate the efficacy of needle aspiration (9,17–19). Gan suggested that TRUS-guided needle aspiration is a feasible alternative to traditional transurethral drainage (17). Collado *et al* reported that a combination of TRUS-guided needle aspiration and adjuvant antibiotic therapy was effective in the treatment of prostatic abscess (18). Aravantinos *et al* and Lim *et al* demonstrated similar results in the treatment of prostatic abscess via TRUS-guided needle aspiration (9,19). In the present case, a 42-year-old diabetic male with CA-MRSA prostate abscess involving the seminal vesicle was successfully treated via adjuvant antibiotics and TRUS-guided prostate needle aspiration.

Prostatic abscess involving the seminal vesicle is a rare disease with potential risk of mortality if the appropriate treatment is not administered, and thus deserves considerable attention. Ultrasound perfusion imaging plays an important role in diagnosing prostatic abscess involving the seminal vesicle. In the present case, early treatment with appropriate antibiotics and TRUS-guided needle aspiration of the prostatic abscess resulted in a shorter hospital stay, and lower risk of local recurrence and mortality.
